# Therapy with new generation of biodegradable and bioconjugate 3D printed artificial gastrointestinal lumen

**DOI:** 10.22038/ijbms.2021.47925.11013

**Published:** 2021-03

**Authors:** Matin Karbasian, Seyed Ali Eftekhari, Mohammad Karimzadeh Kolamroudi, Bahareh Kamyab Moghadas, Peiman Nasri, Amir Jasemi, Mahshid Telloo, Saeed Saber-Samandari, Amirsalar Khandan

**Affiliations:** 1 Department of Mechanical Engineering, Khomeinishahr Branch, Islamic Azad University, Khomeinishahr/Isfahan, Isfahan, Iran; 2 Mechanical Engineering Department, Eastern Mediterranean University, Gazimagusa, TRNC, Via Mersin 10 Turkey; 3 Department of Applied Researches, Chemical, Petroleum & Polymer Engineering Research Center, Shiraz Branch, Islamic Azad University, Shiraz, Iran; 4 Department of Chemical Engineering, Shiraz Branch, Islamic Azad University, Shiraz, Iran; 5 Metabolic Liver Disease Research Center, Isfahan University of Medical Sciences, Isfahan, Iran; 6 Child Growth and Development Research Center, Research Institute for Primordial Prevention of Non-Communicable Disease, Isfahan University of Medical Sciences, Isfahan, Iran; 7 Firoozgar Hospital, Iran University of Medical Sciences, Tehran, Iran; 8 New Technologies Research Center, Amirkabir University of Technology, Tehran, Iran

**Keywords:** Cardiovascular, Gastrointestinal lumen, Magnetite nanoparticle, Polyurethane, Tissue

## Abstract

**Objective(s):**

Many patients die due to vascular, gastrointestinal lumen problems, and coronary heart diseases. Synthetic vessels that are made of biodegradable-nanofiber polymers have significant properties such as proper biodegradability and efficient physical properties such as high strength and flexibility. Some of the best options for supporting cells in soft tissue engineering and design are applications of thermoplastic polyurethane polymer in the venous tissue. In this study, the first nanoparticle-reinforced polymeric artificial prosthesis was designed and tested to be used in the human body.

**Materials and Methods:**

In this study, artificial gastrointestinal lumen were fabricated and prepared using a 3D printer. To improve cell adhesion, wettability properties and mechanical stability of elastin biopolymer with magnetic nanoparticles (MNPs) as well as single-walled carbon nanotubes (SWCNT) were prepared as separate filaments. MNPs were made in 5–7 mm sizes and then examined for mechanical, biological, and hyperthermia properties. Then, the obtained results of the gastrointestinal lumen were simulated using the Abaqus software package with a three-branch. The results were evaluated by X-ray diffraction (XRD) and scanning electron microscopy (SEM) for morphology and phase analysis.

**Results:**

The obtained results of the designed vessels showed remarkable improvement in mechanical properties of the SWCNT vessels and hyperthermia properties of the vessels containing the MNPs. The results of computational fluid dynamics (CFD) analysis showed that the artificial vessels had lower shear stress at the output.

**Conclusion:**

Five-mm MNP containing vessels showed noticeable chemical and biological properties along with ideal magnetic results in the treatment of thrombosis and vascular obstruction.

## Introduction

Tissue engineering is an interdisciplinary science, which is focused on developing and integrating chemistry, physics, mathematics, and biology with engineering and seeks to solve medical problems such as tissue loss and organ failure or provide solutions for them (1-3). Moreover, tissue engineering is a multidisciplinary field of principles and applications of engineering and biological sciences to understand the fundamental relationships between structure and function in natural and diseased tissues. This domain is a combination of cells, engineering, materials, physical, and chemical factors that aims to maintain tissue balance, improve target tissue function, or replace tissue biomarkers. For this reason, using stem-cells is seen in the discussion of regenerative medicine and tissue engineering (4-5). Most tissue engineering definitions cover a wide range of applications, which means applications that repair or replace part or all of the tissues (such as bone, cartilage, blood vessels, bladder, skin, muscle, etc.). Scientists have been able to cultivate cells outside the body for years; however, the technology of developing complex three dimensional (3D) cellular networks for replacing damaged tissues has recently been developed (6-7). In definition, constructing a tissue in an engineering manner requires designing an artificial vessel with the appropriate physical structure, which enables cell adhesion, migration, proliferation, differentiation, and ultimately growth and replacement with new tissue (8-10). According to the surgeons, biomaterials refer to the four properties of biocompatibility and non-toxicity, resistance to infection, preservation of mechanical properties, and the ability to provide specific properties that are necessary for their application in the body (11-13). Biocompatibility and non-toxicity are of interest in this regard. Most tissues require mechanical and structural properties to function properly. The term has also been coined to perform specific biochemical functions using cells in an artificial support system (for example, artificial heart, pancreas, or liver) (14-16). Recent advances in tissue engineering aim to overcome the limitations of conventional organ transplantation and material transplantation methods. In this context, there is a great potential in organ and artificial tissue construction so that transplanted tissue and organs can grow after transplantation in the recipient. With this method, there is a permanent solution to treat damaged tissues. Therefore, there is no need for complementary therapies and as a result, the cost of treatment is greatly reduced. So far, tissue engineering has been used to repair many tissues such as bone, cartilage, blood vessels, and skin (17-21). Hyperthermia was induced locally to treat cancer. Lack of heat distribution in all tumor cells, inadequate amount of heat produced, and unintended heat treatment of healthy cells are the major challenges of current hyperthermia methods. Nanoparticles promise the most effective treatment with the ability to specifically accumulate in tumor tissue as well as the ability to generate more heat. Metal nanoparticles and magnetic nanoparticles are among the most important nanoparticles used in hyperthermia that can be used to treat soft tumors (19-22). To achieve these conditions in tissue engineering, they use cells embedded in an artificial support system. Cells are often implanted or embedded in artificial structures that are able to mimic and support 3D tissue structures. This structure is called artificial blood vessel. Synthetic vessels are obtained by using biodegradable and biocompatible materials (23-31). The artificial blood vessels (ABV) have porosity in their structure, which helps better cell adhesion and placement. The size and intensity of porosity can be controlled. It should be noted that the main part of the work is designing the characteristics of the ABVs, which determine the size of the holes, the intensity of the porosity, and the degree of degradability. Each tissue has its own biological and physical characteristics such as size and shape (32-45). Therefore, in practice, any artificial vessel must be capable of incorporating specific biological and mechanical effects to improve and alter cellular behavior. For this purpose, each artificial vessel is designed based on its target tissue characteristics. Selecting the type and texture of the artificial vessel is the most important part of the work so that the damaged tissue can eventually be replaced. To achieve this, artificial vessels must have a number of structural features. The main purpose of this study was to build a new ABV, which can be used in vascular problems like aneurysm, varicose veins, and coronary artery bypass grafts. In this study, two types of composite blood vessels are introduced using fused deposition modeling (FDM) and mechanical and biological evaluation performed on the samples. Then, computation fluid dynamic (CFD) is used to simulate the minimum and maximum values for the ABV.

## Materials and Methods

In this study, an artificial vessel was made of biodegradable tubular materials that were distributed in a bio-nanocomposites substitute, which was a composite-base for fabrication of polyurethane thermoplastic experiment in this research. This was a self-restoring polymeric biocompatible base. Furthermore, the magnetic material (Fe_3_O_4_) in the bionic composite substrate in the magnetic field was investigated too. 

***Fabrication of composite filler***

Two types of thermoplastic polyurethane (TPU) based filaments with nanocomposite fillers were fabricated. The first filler was fabricated using 90 wt% TPU + 5 wt% elastin + 5 wt% single-walled carbon nanotube (SWCNT) composed with specific acetic acid solvent. The solution was homogenized and stirred for 4 hr on a magnetic stirrer at 50 °C and 400 rpm. Also, similar to filler 1 preparation, A 5 wt% Fe_3_O_4_ (magnetite nanoparticles (MNPs)) was fabricated and stirred on the magnetic stirrer. Finally, both filler 1 solution and filler 2 solution were placed in an ultrasonic bath for 3 hr. Both SWCNT and MNPs were purchased from the Merck Company. The final solution was then poured into an extruded mold and placed in the oven for 60 min at 60–80 °C. Then, filler 1 was removed from the oven and placed at ambient temperature (20–25 °C) and roll the cooled fillers. For chemical and phase analysis, X-ray diffraction (XRD) and scanning electron microscopy (SEM) were used. 

***Tensile strength evaluation***

**Figure 1:**
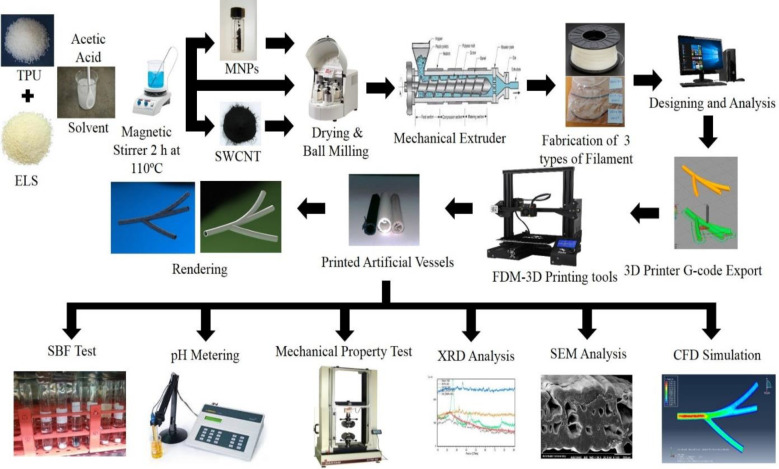
Schematic overview of TPU-elastin-containing single-walled carbon nanotubes and magnetite nanoparticles

**Figure 2:**
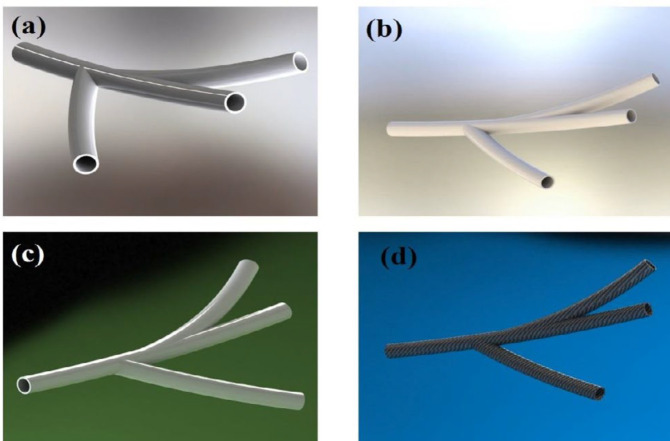
Schematic of the design and fabrication of a bi-nanocomposite artery made by 3D printing

To obtain the value of the mechanical properties of the 3D-printed artificial tissue, elastic modulus was used. The specimens were examined at the Amirkabir University of Technology using tensile tests (SANTAM, STM-50) with a tensile strength of 0.5 (mm/min). The specimens made of 50 mm length and 18.85 mm cross-section were attached to the two ends of the tensile test machine. Figure 1 shows the schematic preparation of artificial tissue using a 3D printing technique. Proportional to the cross-sectional area of ​​the specimens, the distance between the two clamps was measured, which was the starting point of the stretch, and was eliminated for the calculations. Figure 2 shows the initial design of the artificial using the SolidWorks software package before applying it in the simplified 3D software. All 7 specimens were stretched until the vessel layers were ruptured. The output of the device, in the form of force and displacement data, were converted to stress and strain by holding the diameter and initial length of each specimen. Finally, using the elastic region gradient of the stress-strain diagram, the elastic modulus of each sample was obtained. According to the standards of American Society of Testing Materials, the tube must be taken to examine the mechanical properties of the bi-axial tension test. Table 1 indicates the mechanical properties of the artificial tissue for CFD simulation such as Poisson ratio, elastic modulus, and porosity value.

***Characterization of artificial tissue***

*XRD analysis*

   To determine the phases present in the synthesized powder and synthetic composite vessels, XRD and PHILIPS PW3040 tests (at Amirkabir University of Technology - Advanced Central Laboratory) were used, and the scanning angle (2θ) ranged from 10 to 90 ° beneath 40 kV and 30 mA.

*SEM analysis*

    In order to study the size and morphology of the pores and porosity in the artificial tissue, scanning electron microscopy (AIS2100, SERON TECHNOLOGIES Co., South Korea) was used (magnification 15 to 300,000 times, resolution 3.54 nm, electron acceleration voltage 0.5 kV to 30 kHz, image; SEI, Secondary electron image; tungsten filament, detector; secondary electron detector, located at Amirkabir University of Technology) to enhance the electrical conductivity and resolution of the specimens and images, a thin layer of gold (Au) was sprayed on the specimens prior to the imaging.

*Porosity evaluation*

In this study, the artificial tissue was examined for porosity value using Image-J software and SEM images. The SEM images of the artificial tissue inserted into the Image software and the open porosity of the tissue were investigated (27, 28). 

***Hyperthermia evaluation***

Hyperthermia test was performed in ethanol solution as neutral fluid in the solenoid circuit for 0–15 sec at 38-42 °C. The obtained results were plotted as a temperature-time diagram. The obtained results were presented where the specimens must have average temperature variations in order to have a suitable thermal and heat effect on the artificial tissue. 

***CFD analysis of blood flow***

In this study, the Abaqus software, as a powerful finite element software, has proven its ability to simulate and analyze fluid problems. The existence of computational fluid dynamics (CFD) solver allows the user to achieve precise solutions to various problems in the field. A CFD simulation examines various fluid parameters such as velocity, pressure, density. The basic structure of heat and fluid transfer problems govern equations that are directly derived from the laws of survival of the physical properties of the fluid. Naiver-Stokes equations are regarded as the mathematical model for physical phenomena. The cases and parameters presented in blood mechanical properties were extracted from the article by Zhao *et al.* (23). The simulation assumed a blood fluid viscosity of 2.5 E^-9^ (MPa) and a blood density of 1 (Kg/mm^3^). The vessel was designed in three branches and blood was assumed to be a homogeneous fluid material. Due to the simulation process in the first stage, the flow was investigated as one input and three outputs. Abaqus analytical software used the General Flow solver to solve the process. The process timeout assumed 1 sec. The rate of blood inlet velocity was set to 0.1 (m/s) and the amplitude of the onset of flow was considered tabular and linear. To create a direct flow between the branches of the vessel, the output pressure was set at 0 MPa to move the blood in the vessel without applying opposite pressure. To create boundary conditions, the vessel walls were defined for non-slip software, and for the whole model, the flow rate for all speeds was set to 0 (mm/s) for the initial fluid input mode in which the vessel stenosis was considered TET structure. The vessel diameter was selected as 1 mm in diameter. The mesh length was set to 0.3 and after all initial analysis, steps were adjusted. The results of the analysis in the three-branch vessel showed the highest applied pressure of 2.187 E^-8^ MPa, which were regularly discharged from the three branches with different velocities and pressure.

## Results

**Figure 3:**
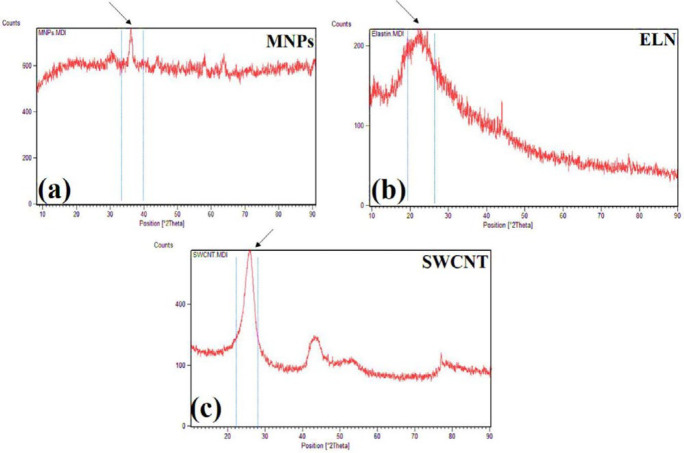
X-ray diffraction of (a) magnetic nanoparticles, (b) elastin powder, and (c) single-walled carbon nanotubes in pure form

MNPs: magnetic nanoparticles; ELN: Elastin; SWCNT: single-walled carbon nanotubes

**Figure 4:**
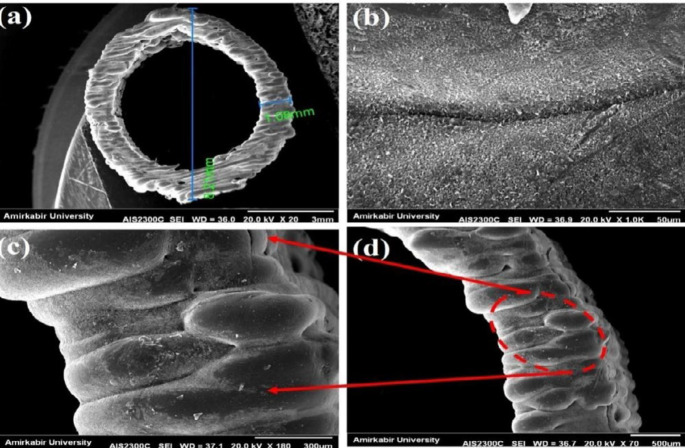
SEM images (a) Artificial vein thickness, (b) Highly scaled sample surface, (c) Triangular melt sample surface, and (d) Vessel thickness of approximately 1000 microns equivalent to 1 mm of specimen

**Figure 5:**
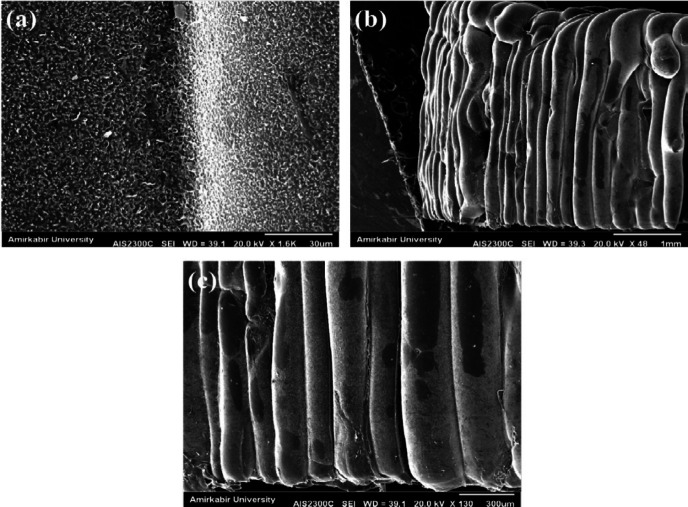
SEM images (a) Artificial vessel thickness, (b) Low-scaled sample surface, (c) Triangular melted specimen surface made by 3D printing of different surfaces after immersion in simulated body solution

**Figure 6:**
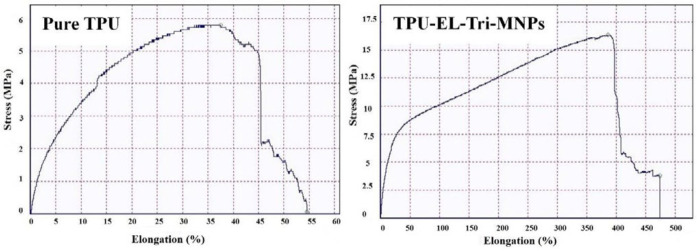
Stress-elongation diagram of the prepared vessel (a) Raw TPU material and (b) composite triangular-shaped specimen containing prepared MNPs

**Figure 7:**
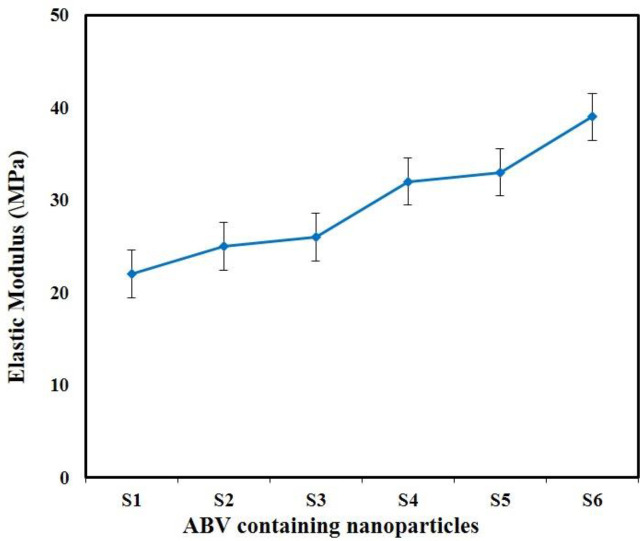
Elastic modulus of the prepared sample containing MNPs and SWCNT with different diameters

MNPs: magnetic nanoparticles; SWCNT: single-walled carbon nanotubes

The obtained results showed that the samples have variable phase variations, which had a relationship with each other. Specimens made of polyurethane thermoplastic-elastin compound with different weight percentages of MNPs and SWCNT were produced. As shown in Figure 3, the XRD pattern peaks of MNPs without impurity were observed. Since the XRD patterns of the MNPs phases were very close together, the composite samples may be a combination of MNPs phases. Due to the high saturation magnetism of the sample containing MNPs and its wide peaks in the composite structure, it can be determined that the non-magnetic phases within the samples were very small. The morphology of the bionic-sedimentary samples made by fusion-deposition by SEM is shown in Figure 4. These images show that the vessels made by the FDM method are 1 to 2 mm in thickness. The vessels had interconnected surfaces with very low porosity, and these specimens had triangular and square structures. The specimens were prepared with a triangular structure, higher interconnectivity, and low porosity rate. The obtained results for porosity and fusion integrity of the filaments can reduce permeability and increase surface adhesion. Figure 4 (a-d) shows the SEM images of the samples containing thermoplastic base polymers and elastin, which were uniformly joined. The obtained results show the thickness of the artificial vessel, the surface of the specimen with high scaling, the fused surface of the specimen with triangular structure, and the thickness of the vessel, which was about 1000 microns, equivalent to 1 mm. Different weight composition of the MNPs materials and SWCNT are not visible in the microscopic images. Longitudinal uniaxial tests were performed to estimate and evaluate the mechanical properties (tensile strength) of the artificial vessel made by 3D printing. Then, the tensile strength and tensile modulus of these vessels were obtained, which was equivalent to the slope of the stress-strain curve. The mean thickness of the specimens was 5–7 mm. As shown in Figure 5, a 100-micron precision 3D printer was used to make such artificial vessels. However, composite filaments were used instead of single filaments. Since the function of the prosthetic after implantation is highly dependent on its adaptation to normal tissue, controlling the structure and material arrangement of the 3D printer can increase the prosthetic adaptability to the actual vessel. Figure 6 shows the stress-strain diagram of the longitudinal bending test for the specimens. The maximum elastic modulus was extracted from the area with maximum stress-strain before failure. Alteration of mechanical properties of the vessels is an important factor in determining and evaluating changes in cardiovascular features due to age and disease from atherosclerosis investigation. The applications of this artificial tissue are in vascular grafts, angioplasty, and bypass surgery (46-56). Moreover, designing artificial tissues and biological systems of vascular elongation in tissue engineering are highly dependent on the mechanical properties of these tissues. Figure 7 show that SWCNT has approximately 3-fold yield stress. However, as shown in Figure 7, the yield stress of the sample containing MNPs is lower than that of the SWCNT sample. Normally, human veins have a peripheral tensile strength of about 25.3 MPa to 29 MPa. The tensile strength of this artificial vessel composite is one of the most important features when evaluating the efficacy and quality for soft tissue replacement. Mechanical performance is the most important factor in maintaining and stabilizing ABV after being implanted in the *in vitro* environment because as soon as the natural vessel is replaced. This new tissue is affected by different loads from different directions and as these mechanical loads are not tolerated, the tissue may encounter ruptures after a short time. This strength is related to several factors such as the matrix of the material and matrix strength, type of reinforcing, mineral particles, size, interaction between the organic component and mineral sediments, and the ratio of these components in a composite. Tensile testing can determine the initial strength of ABV to some extent. However, ABV is placed in the inner environment due to deposition on the surface and mineralize the surface over time. This tensile strength of ABV is related to several factors such as the matrix material and the type of reinforcing, mineral particles, and the size of nanomaterials. Tensile testing can partially determine the initial strength of the artificial veins. However, artificial arteries become stronger over time due to deposition on the surface and mineralization of the surface as time passes. In the elastic zone that has a direct strain proportional to the stress with increasing stress, the strain also increases linearly. The area of ​​compression occurs after the initial drop in the stress value. The elastic modulus (slope of the initial linear part of the graph) and the tensile strength (maximum stress) of the specimens are shown in Figure 7. 

## Discussion

**Figure 8:**
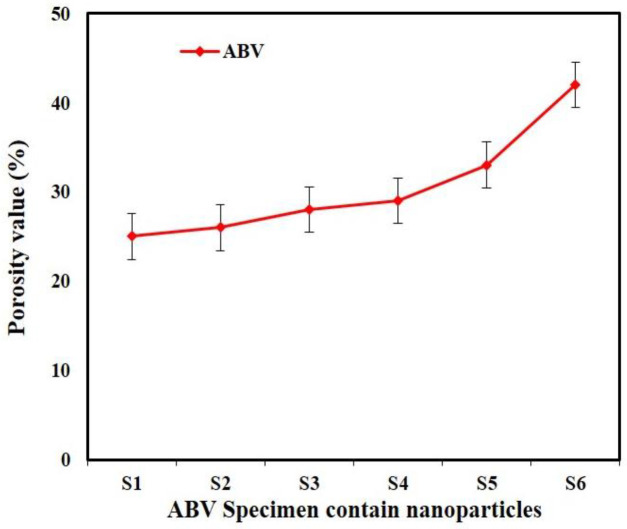
Porosity analysis of artificial tissue using Image-J software and SEM images

**Figure 9:**
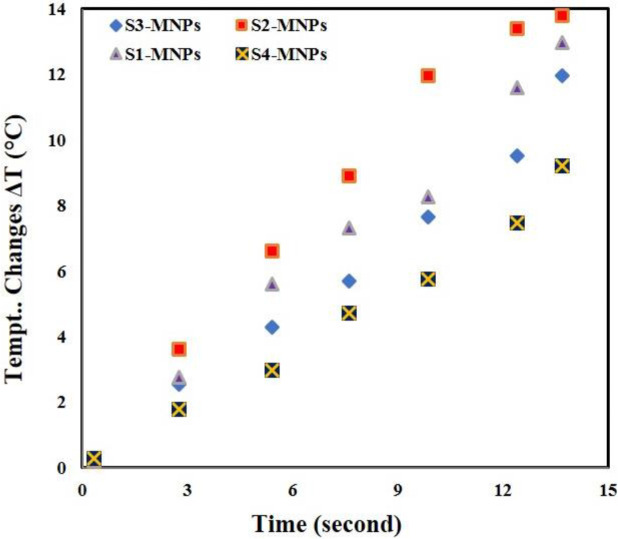
Results of a 100 kHz hyperthermia test at -15 °C for 15–10 sec in ethanol solution in a solenoid loop for triangular and square samples containing MNPs

**Figure 10:**
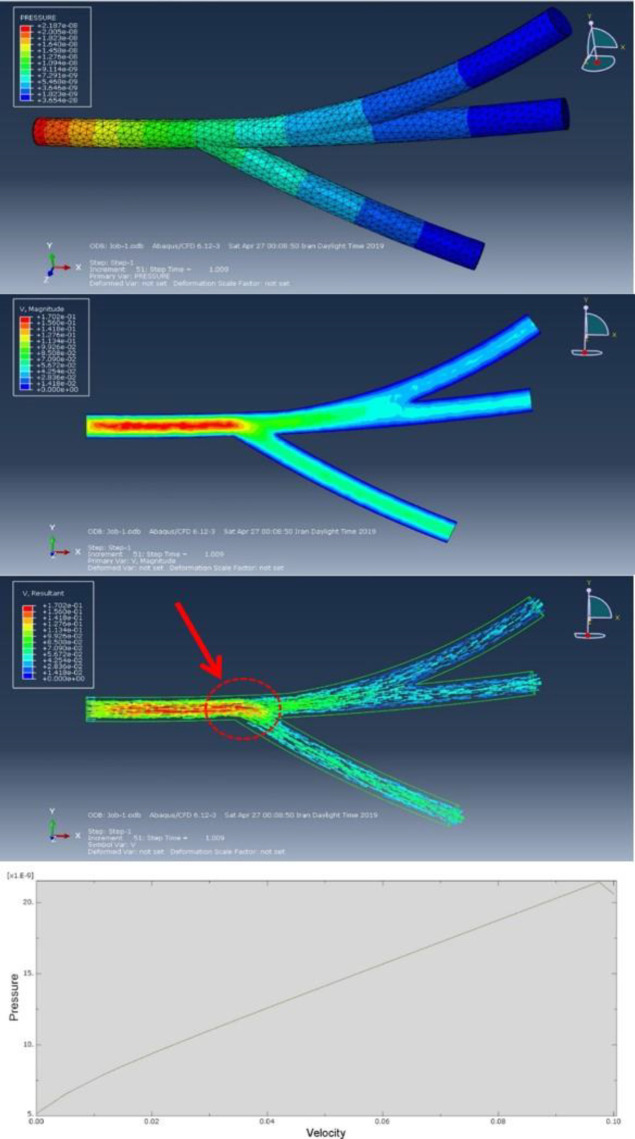
CFD analysis of veins in the Abaqus software with the blue dots

Figure 8 shows the porosity evaluation, as shown by increasing the SWCNT the porosity percentage increased and its pore size decreased. Increasing and decreasing the amount of SWCNT increased tensile strength and porosity. The cause of this phenomenon can be attributed to the special properties of carbon fibers in the presence of any matrix material. Proper insights into the molecular mechanisms can also lead to understanding hyperthermia-related therapies such as temperature-sensitive nanomaterials or drug delivery sensitiveness beside temperature. To investigate the magnetic and hyperthermia behavior of 3D nanocomposite artificial arteries, the sample containing magnetic nanoparticles with triangular and square structures are shown in Figure 9. According to Figure 9, triangular-shaped specimens exhibit better hyperthermia properties in saturated magnetic signals. The magnetic properties of sample 1 and sample 3 indicate a lack of unsuitability and survivability, also indicating ferromagnetic properties. The mechanical energy was generated by ultrasound through friction, which then was converted to thermal energy. Figure 9 illustrates the function of hyperthermia to eliminate annoying tissue in the blood vessel. The simulation according to the results of the analysis of blood flow through an artificial tissue is presented in Figure 10. The following images illustrate the pressure contour in the CFD analysis of the blood flow in the vein by Abaqus in the CFD environment. In the initial CFD study, all outputs were open and the highest pressure and stress was obtained in the marked area as shown in Figure 10. 

**Figure 11:**
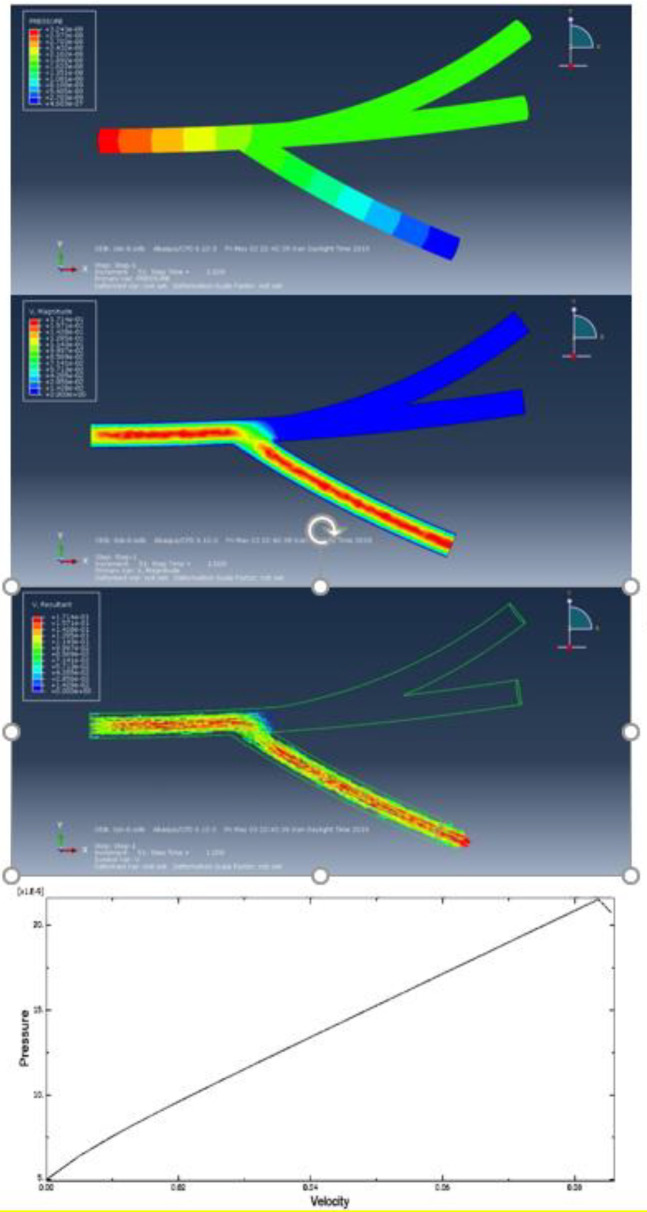
CFD analysis of veins in the Abaqus software with the blue dots as one of the outputs is open

Figure 11 shows CFD simulation of the tissue with open and closed branches along with the results of pressure-based blood velocity analysis in the turbulent area. Figure 11 shows that due to the steeper slope of output 3 of the tissue in the pressure-velocity diagram, a relative increase can be seen in maximum pressure in the occipital region and a relative decrease in the flow velocity corresponding to the maximum pressure in the occipital region compared with output 1.

TPU:thermoplastic polyurethane; CFD: computational fluid dynamics

**Table 1:** Mechanical properties of produced artificial tissue fabricated using 3D Printing

**Sample**	**Poisson ratio**	**Elastic modulus (MPa)**	**Porosity (%)**
**S1-TPU-ELN**	0.29	22	25
**S2-MNPs-**	0.29	25	26
**S3-MNPs-∆**	0.28	26	28
**S4-SWCNT-**	0.31	32	29
**S5-SWCNT-∆**	0.31	33	33
**S6-7 mm**	0.30	39	42

TPU:thermoplastic polyurethane; SWCNT: single-walled carbon nanotubes; MNPs: magnetic nanoparticles

SEM: scanning electron microscopy

SEM: scanning electron microscopy

TPU:thermoplastic polyurethane; MNPs: magnetic nanoparticles

SEM: scanning electron microscopy

MNPs: magnetic nanoparticles

CFD: computational fluid dynamics

CFD: computational fluid dynamics

## Conclusion

In this study, for the first time, a synthetic polymer reinforced with SWCNT and MNPs was fabricated for application in the human body. Artificial tissues are made of biodegradable polymer nanofibers with notable physical properties such as efficient tensile strength and proper flexibility. The results of the investigated tissue showed significantly improved mechanical properties. Furthermore, changing the size of the vessels increased mechanical properties such as elastic modulus and tensile strength. The obtained results indicated that the elastic modulus increased from 25 MPa to 26 MPa. These samples had the lowest amount of weight loss, pH changes, and slight changes in the SBF and PBS solution. The results of the CFD analysis showed that the artificial tissue had lower shear stress at the output. Finally, a vessel containing 5-mm MNPs can be presented as a suitable choice with appropriate chemical and biological properties for treating tissue disease in atherosclerosis and gastrointestinal lumen for children.
